# Use of a Genetic Variant Related to Circulating FXa (Activated Factor X) Levels to Proxy the Effect of FXa Inhibition on Cardiovascular Outcomes

**DOI:** 10.1161/CIRCGEN.120.003061

**Published:** 2020-08-13

**Authors:** Dipender Gill, Stephen Burgess

**Affiliations:** 1 Department of Epidemiology and Biostatistics, School of Public Health, Imperial College London, United Kingdom (D.G.).; 2 Medical Research Council Biostatistics Unit, Cambridge Institute of Public Health (S.B.).; 3 Cardiovascular Epidemiology Unit, Department of Public Health and Primary Care, University of Cambridge, United Kingdom (S.B.).

**Keywords:** cardiovascular disease, coronary artery disease, Mendelian randomization analysis, serine protease

Coagulation FX (factor X) is a serine protease that catalyzes the formation of fibrin clots. Although this maintains hemostasis, it can also result in pathological thrombi and emboli.^1,2^ FXa (activated FX) inhibitors are efficacious for preventing deep venous thrombosis, pulmonary embolism,^1^ and cardioembolic stroke related to nonvalvular atrial fibrillation.^2^ However, the efficacy of FXa inhibitors for other forms of cardiovascular disease is not known.

Genetic variants related to circulating levels of a coagulation factor can be used as instrumental variables in Mendelian randomization analyses to study the effects of drugs that inhibit that coagulation factor.^3^ The aim of this work was to employ a genetic instrument for circulating FXa levels in an exploratory investigation into the effects of varying FXa levels on cardiovascular outcomes.

As the instrument for circulating FXa levels, we used the rs61753266 variant in the *F10* gene that has been associated with plasma FXa levels at *P*=8×10^-15^ in a study of 3301 European-ancestry individuals.^4^ Although the rs547138 variant in the *F10* gene was associated with plasma FXa levels at *P*=6×10^-22^ in the same study, it was also associated with Protein Z–dependent protease inhibitor at *P*=5×10^-4 4^. Given the role of Protein Z–dependent protease inhibitor in inhibiting FXa, this likely represents a pleiotropic association that could bias Mendelian randomization analyses investigating the effect of FXa, and therefore this variant was not included. The rs61753266 variant we used had a weaker association with circulating Protein Z–dependent protease inhibitor (*P*=0.05).

We used the UK Biobank to perform analyses, considering the outcomes of coronary artery disease (CAD), peripheral artery disease, ischemic stroke, intracerebral hemorrhage, subarachnoid hemorrhage, deep vein thrombosis, and pulmonary embolism. Genetic association estimates for these outcomes were obtained from 367 570 unrelated European-ancestry participants. Cases were defined based on *International* *Classification of Diseases*, *Ninth and Tenth Revisions*, Office of Population Censuses and Surveys Classification of Surgical Operations and Procedures (fourth revision), and participant self-reported data (Table). Only incident cases recorded until October 11, 2019 were considered. Relevant ethical approval for the UK Biobank study was obtained from the North West Multicentre Research Ethics Committee, and all participants provided informed consent. UK Biobank data are available on request (see the Acknowledgments section). The statistical code used is available from the corresponding author upon reasonable request.

**Table.**
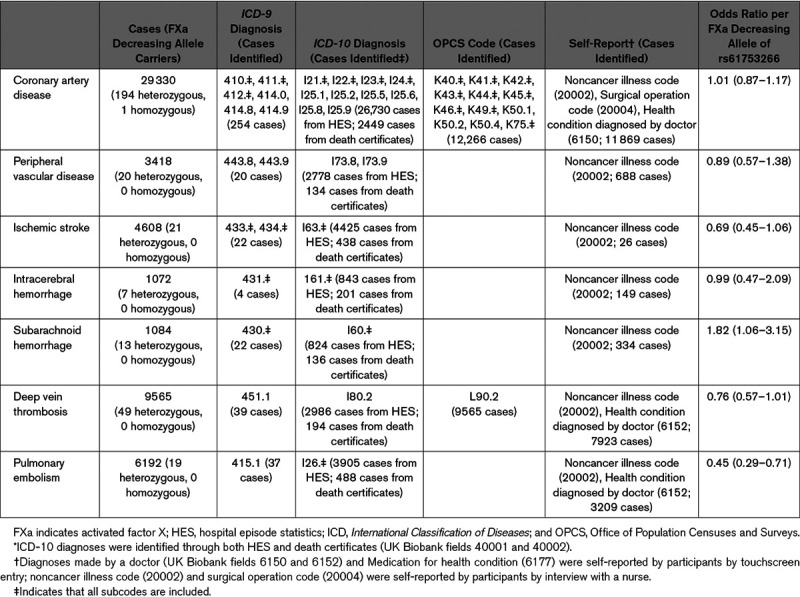
Associations of the Genetic Instrument for FXa Levels (rs61753266) With Cardiovascular Outcomes in the UK Biobank

Odds of each outcome per FXa decreasing allele of rs61753266 (frequency 0.3%) are detailed in the Table. Consistent with existing randomized-controlled trial data, the genetic instrument for lower FXa levels was associated with reduced deep vein thrombosis and pulmonary embolism risk.^1^ This instrument is also associated with higher subarachnoid hemorrhage risk, in keeping with the increased bleeding risk associated with FXa inhibitors. These results, therefore, serve as a form of positive control, supporting the validity of the variant as an instrument for FXa inhibition. There was some suggestion that the genetic instrument for lower FXa levels may be associated with a reduced risk of ischemic stroke and peripheral artery disease, although the 95% CIs were broad. Inhibitors of FXa are known to be effective for reducing the risk of stroke related to nonvalvular atrial fibrillation.^2^ The point estimates for the associations of the FXa lowering variant with CAD and intracerebral hemorrhage risk were close to the null.

This work represents an efficient way to explore the potential clinical applications of FXa inhibitors. The use of a genetic instrument for FXa levels helps overcome environmental confounding and reverse causation bias to allow causal inferences to be drawn. However, the approach also has limitations. Although the location of the variant we employ at the *F10* locus and its association with plasma FXa levels both support its validity as an instrument for FXa levels,^4^ we cannot exclude the possibility that it affects risk of the considered outcomes through pathways independent of FXa levels, to bias the results of our analysis. Furthermore, as apparent from the confidence intervals of the results, our study had limited statistical power. Given the previously described association between our instrument for circulating FXa levels and CAD,^5^ and the role of FXa in inflammation, vascular remodeling, and fibrosis, our null finding for this outcome should be interpreted with caution. The discrepancy may, in part, be attributable to the criteria used to diagnose CAD—although Paraboschi et al^5^ considered angiographically confirmed cases and controls with no angiographic evidence of coronary atherosclerosis, our current study used *International Classification of Diseases* and Office of Population Censuses and Surveys codes, and self-report for case ascertainment (Table), with noncases considered as controls.

In conclusion, the findings of this Mendelian randomization study are consistent with the results of clinical trials in supporting an effect of FXa inhibition on reducing risk of venous thromboembolism and increasing risk of subarachnoid hemorrhage. The results did not support that FXa inhibition is associated with reduced CAD risk in a general population. Although further study is required to investigate the discrepancy with previous work,^5^ this information may be used to help prioritize future clinical trials.

## Acknowledgments

This research has been conducted using the UK Biobank resource (application 29202). The UK Biobank data is available on application (http://www.ukbiobank.ac.uk/register-apply).

## Sources of Funding

Dr Gill is funded by the Wellcome Trust 4i Programme (203928/Z/16/Z) and British Heart Foundation Centre of Research Excellence (RE/18/4/34215) at Imperial College London. Dr Burgess is supported by Sir Henry Dale Fellowship jointly funded by the Wellcome Trust and the Royal Society (204623/Z/16/Z). The funding sources had no role in the design, acquisition of data, analysis, interpretation, or write up of this study.

## Disclosures

Dr Gill is employed part-time by Novo Nordisk. The other author reports no conflicts.

## References

[1] Lassen MR Gallus A Raskob GE Pineo G Chen D Ramirez LM; ADVANCE-3 Investigators. Apixaban versus enoxaparin for thromboprophylaxis after hip replacement. N Engl J Med. 2010;363:2487–2498. doi: 10.1056/NEJMoa1006885 21175312 10.1056/NEJMoa1006885

[2] Patel MR Mahaffey KW Garg J Pan G Singer DE Hacke W Breithardt G Halperin JL Hankey GJ Piccini JP; ROCKET AF Investigators. Rivaroxaban versus warfarin in nonvalvular atrial fibrillation. N Engl J Med. 2011;365:883–891. doi: 10.1056/NEJMoa1009638 21830957 10.1056/NEJMoa1009638

[3] Gill D Georgakis MK Laffan M Sabater-Lleal M Malik R Tzoulaki I Veltkamp R Dehghan A Genetically determined FXI (Factor XI) levels and risk of stroke. Stroke. 2018;49:2761–2763. doi: 10.1161/STROKEAHA.118.022792 30355187 10.1161/STROKEAHA.118.022792

[4] Sun BB Maranville JC Peters JE Stacey D Staley JR Blackshaw J Burgess S Jiang T Paige E Surendran P Genomic atlas of the human plasma proteome. Nature. 2018;558:73–79. doi: 10.1038/s41586-018-0175-2 29875488 10.1038/s41586-018-0175-2 PMC6697541

[5] Paraboschi EM Khera AV Merlini PA Gigante L Peyvandi F Chaffin M Menegatti M Busti F Girelli D Martinelli N Rare variants lowering the levels of coagulation factor X are protective against ischemic heart disease. Haematologica. 2020;105:e365–e369. doi: 10.3324/haematol.2019.237750 31699787 10.3324/haematol.2019.237750 PMC7327651

